# “Plutchik”: artificial intelligence chatbot for searching NCBI databases

**DOI:** 10.5195/jmla.2018.500

**Published:** 2018-10-01

**Authors:** Shannon Bohle

**Affiliations:** Owner and President, Archivopedia, Lima, OH 45805

## Abstract

As genetic testing gains ground in medicine, the ability to search across the suite of biomedical and clinical care databases offered through the National Library of Medicine/National Center for Biotechnology Information (NCBI)—such as PubMed, GENE, Structure, the Genetic Testing Registry, and others—holds the potential to enhance quality of clinical care best practices. “Plutchik” is a voice-enabled, embodied artificial intelligence (AI) chatbot that can perform highly technical medical searches in and across the NCBI suite of databases.

A chatbot is a computer program designed to interact with people by closely emulating human conversation, as idealized in the Turing Test for artificial intelligence (AI) [1, 2]. The “Plutchik” AI medical chatbot (and avatar robot) is a virtual-world testbed that explores two major emerging therapeutic technologies: AI-driven gene editing and drug discovery. Many of the National Center for Biotechnology Information (NCBI) databases hosted by the National Library of Medicine (NLM) contain sequenced genomes and genomics-related information [3]. The methods by which AI has already been used to help create gene-edited therapies, some using NCBI tools such as RefSeq, extend beyond the scope of this article [4].

Named after Robert Plutchik (1927–2006), “Project Plutchik” aims to create an “empathetic” embodied AI chatbot to search, retrieve, analyze, and communicate medical information and to interact with health care providers in natural language and “voice” using 3D facial expressions and gestures. “Project Plutchik” is the first chatbot available to the public (subject to licensing conditions) to search data from the NCBI databases by diagnostic International Classification of Diseases, Tenth Revision, Clinical Modification (ICD-10-CM) codes and to retrieve the information in voice. These code numbers are commonly used to classify and report medical diagnoses and hospital inpatient procedures. This process holds the potential to improve clinical care and enhance and streamline health information services that libraries provide.

In addition to the “Plutchik” bot’s search-and-retrieval feature, it was recently programmed to deliver a fully automated slideshow lecture about NASA’s genomic study of twin astronauts Mark and Scott Kelly for National DNA Day (25 April 2018) in association with the National Space Society’s activities in the Second Life virtual world [5]. The AI chatbot delivered the lecture in voice, changed slides, initiated videos, and answered questions following the talk, as well as integrated voice, emotive gestures, and facial expressions, and 3D learning objects. This feature could be valuable to libraries through automated delivery of library instruction and has the potential to enhance user engagement and learning, as well as provide a cost-effective solution for delivering information about library services and materials.

“Project Plutchik” is the successor to an earlier effort called “Curiosity AI,” a Department of Defense award–winning project by the author of this article [6]. This initial program explored the potential of AI on Mars, using a variety of simulated robots to perform specialized tasks (Intelligent Life Beyond Earth and Satellites Demonstrated in Second Life). One aspect of the “Curiosity AI” program that was particularly relevant to libraries was its innovative incorporation of a humanoid 3D AI-driven avatar to retrieve links from library literature databases using natural language queries. This unique program along with a few other early implementations, like “Emma the Catbot” at the Mentor Public Library (now defunct) and “Pixel“ at University of Nebraska–Lincoln, were among the first AI chatbots that librarians created to answer questions and retrieve information [7].

Both “Curiosity AI” and “Project Plutchik” for use in Second Life utilize customized programming: artificial intelligence markup language (AIML), Linden scripting language (LSL), and C# programming found in an existing third-party open source viewer. The “Curiosity AI” project ended after pushing that existing viewer’s capabilities to its limits. To enable new features, like voice capability, “Project Plutchik” needed to modify its C# coding, as well as add new LSL scripting and more original AIML files. The official Second Life Viewer is proprietary and cannot be modified. As “Project Plutchik” begins to engage in higher-level analysis through machine learning, particularly deep learning, TensorFlow, an open source software library for machine intelligence, and R, a programming and data visualization language, will be examined for possible future implementation. Whereas “Curiosity AI” operated on random access memory (RAM) only, “Project Plutchik” will require an added backend database to accommodate long-term memory storage and learning algorithms.

According to a 2017 Gartner report, “Over the next few years, conversational interfaces based on natural-language interfaces will become the main design goal for user interaction,” where the goal will be to “create a more natural and immersive user experience by deploying, where effective, conversational platforms and virtual, augmented and mixed reality” [8]. Novel methods of interacting with and training an AI/virtual robot are currently being explored in “Project Plutchik.” A touchpad/touch screen interface has been designed to allow real-time learning that links to the chatbot and will later be connected to a neural network, opening the door for interactivity with mobile phone apps and robotic- or human-wearable touch-sensitive synthetic skin [9].

“Plutchik” is an example of how libraries can use AI to remain relevant to younger researchers who are used to commercial mobile and virtual AI technologies like Siri, Google Assistant, and Alexa [10]. Mobile apps for students and professionals in biomedical, health care, and pharmaceutical-related professions, as well as integration into online medical library services, are the visionary end-goals of this AI project.
